# Surface Landau levels and spin states in bismuth (111) ultrathin films

**DOI:** 10.1038/ncomms10814

**Published:** 2016-03-11

**Authors:** Hongjian Du, Xia Sun, Xiaogang Liu, Xiaojun Wu, Jufeng Wang, Mingyang Tian, Aidi Zhao, Yi Luo, Jinlong Yang, Bing Wang, J. G. Hou

**Affiliations:** 1 Hefei National Laboratory for Physical Sciences at the Microscale and Synergetic Innovation Center of Quantum Information & Quantum Physics, Key Laboratory of Strong-Coupled Quantum Matter Physics (CAS), University of Science and Technology of China, Hefei, Anhui 230026, China

## Abstract

The development of next-generation electronics is much dependent on the discovery of materials with exceptional surface-state spin and valley properties. Because of that, bismuth has attracted a renewed interest in recent years. However, despite extensive studies, the intrinsic electronic transport properties of Bi surfaces are largely undetermined due to the strong interference from the bulk. Here we report the unambiguous determination of the surface-state Landau levels in Bi (111) ultrathin films using scanning tunnelling microscopy under magnetic fields perpendicular to the surface. The Landau levels of the electron-like and the hole-like carriers are accurately characterized and well described by the band structure of the Bi (111) surface from density functional theory calculations. Some specific surface spin states with a large g-factor are identified. Our findings shed light on the exploiting surface-state properties of Bi for their applications in spintronics and valleytronics.

## Abstract (web summary)

The electronic surface states of bismuth present promise for applications exploiting spin and valley degrees of freedom however their study is inhibited by bulk states. Here, the authors directly access the surface-state Landau levels of ultrathin bismuth films under a perpendicular magnetic field.

## Graphical abstract

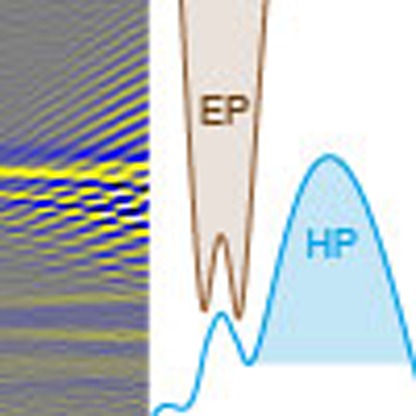

Two-dimensional (2D) electron materials with honeycomb lattice structure commonly have a band structure with the conduction and valence-band edges at degenerate extrema in momentum space, usually referred to as valleys. The valley degree of freedom of electrons, in addition to the intrinsic charge and spin, has potential to be used as an information carrier in next-generation electronics^1^,^2^,^3^,^4^,^5^,^6^,^7^,^8^,^9^,^10^,^11^ and optoelectronics^12^,^13^,^14^,^15^,^16^,^17^,^18^. Among a variety of appealing materials, bismuth (Bi) has attracted particular attention because of its unique electronic properties both in bulk^10^,^11^ and surfaces^19^,^20^,^21^,^22^,^23^,^24^,^25^,^26^,^27^,^28^,^29^. As a heavy element, Bi possesses metallic and spin–split Rashba surface states^19^,^20^ with a vortical spin texture^21^,^22^, resulting from the strong spin-orbit coupling (SOC) with the broken space-inversion symmetry^20^. Due to the rhombohedral symmetry in Bi crystal, the Bi (111) surface has a bilayer-terminated structure^19^, forming a buckled honeycomb lattice that leads to valleys in surface-band structure, which is very similar to many other 2D valley materials with honeycomb lattice^1^,^2^,^3^,^4^,^5^,^6^,^7^,^13^,^14^,^15^,^16^,^17^,^18^. In addition, it has also attracted increasingly study interest in Bi films or bilayers both theoretically^30^,^31^,^32^ and experimentally^33^,^34^,^35^,^36^, owing to the interesting one-dimensional topological edge state, that is, quantum spin Hall state^37^,^38^.

To explore the applications of surface-state valleys, it demands robust valley properties in a material. Theoretical calculations suggested the transition of Bi (111) bilayers going from semiconductor to semimetal with the increase of thickness with the crossover thickness around four or five bilayers^31^,^39^. Bi (111) films with thickness over four bilayers exhibit surface states^39^. The surface-state bands near the Fermi level (*E*_F_) form electron pockets (EPs) around the 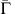 point and the 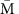 point, and the hole pockets (HPs) along the 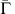
→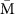 directions in films thicker than 10 bilayers^39^. These multivalley states were characterized in Bi films and Bi crystal by angle-resolved photoemission spectroscopy (ARPES)^21^,^22^,^23^,^24^,^25^,^26^,^27^. Their significant contributions to the conductance were also observed in Bi (111) thin films by the transport measurements^28^,^29^. However, the intrinsic electronic transport properties of Bi (111) surface have not been fully determined. For instance, it was reported that the films thicker than six bilayers show thickness-dependent conductivity, which is ascribed to a coherent bulk-surface coupling^29^. To understand the valley properties of Bi (111) surface for its potential applications in valleytronics^1^,^2^,^3^,^4^,^5^,^6^,^7^, it is necessary to single out the electronic transport properties of the surface states. The measurements of the Landau levels (LLs) of the surface-state EPs and the HPs, especially for the relatively complicated surface-band structures of Bi surfaces^19^, can help to determine the contribution of the metallic surface states to the conduction.

We here focus our investigation on the surface-state valleys of Bi (111) surface. We fabricate ultrathin Bi (111) films with various thicknesses grown on Si (111)-(7 × 7) and measured under the magnetic field using scanning tunnelling microscopy (STM) and spectroscopy (STS). This enables us to unambiguously identify the LLs of the surface states of the Bi (111) surface. Our experimental results are in good agreement with the density functional theory (DFT) calculations. Moreover, it is observed that the out-of-plane spin states undergo spin polarization as the result of the Zeeman splitting, which may further cause the lift of the degeneracy of certain surface-state valleys.

## Results

### STM and STS measurements of a film with thickness of 4.0 nm

**Figure 1:**
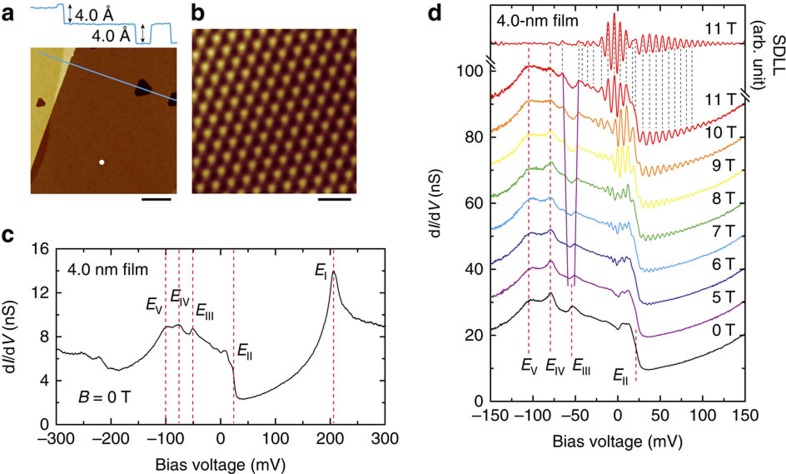
Electronic properties and Landau levels of Bi (111) surface. (**a**) A large scale STM image (acquired at −500 mV and 10 pA), scale bar, 100 nm. The line profile shows the step height of 4.0 Å for the Bi bilayer structure. (**b**) Atomically resolved STM image (acquired at −50 mV and 500 pA) of Bi (111) film of 4.0 nm thick, scale bar, 1 nm. (**c**) Typical d*I*/d*V* spectrum obtained at centre regions of a large terrace (white dot in **a**), acquisition conditions: −300 mV and 4.0 nA, with a sinusoidal modulation of 2 mV by root mean square (r.m.s.), at magnetic field strength *B*=0 T. The red dashed lines indicate the energy positions of several features, labelled as *E*_*i*_ (*i*=I, II…, V). (**d**) d*I*/d*V* spectra recorded in different magnetic field strength, acquired at −150 mV and 4.0 nA with a sinusoidal modulation of 0.5 mV (r.m.s.). All of the spectra are averaged over 10 repeated measurements. The topmost spectrum is the second derivative of the Landau-level (SDLL) spectrum obtained at 11 T, where the LL peaks are more clearly shown by eliminating the background in the d*I*/d*V* spectrum. The spectra are shifted vertically for clarity. The pink lines around the *E*_III_ indicate the peak splitting with the increase of the field. The black dashed lines indicate the peak positions in the SDLL spectrum corresponding to the ones in the d*I*/d*V* spectrum.

Figure 1a shows a representative large-scale image of a 4.0-nm-thick Bi film grown on Si (111)-(7 × 7). The film has a typical step height of about 4.0 Å and the hexagonal structure with the lattice constant of about 4.5 Å (Fig. 1b), indicating its orientation along the (111) direction^19^,^40^. Figure 1c shows a typical d*I*/d*V* spectrum obtained from the 4.0-nm film. Several features are labelled as *E*_*i*_ (*i*=I, II…, V) according to their energy positions. Figure 1d gives the d*I*/d*V* spectra from the film measured at different magnetic field strengths. The field is perpendicular to the sample surface. The LLs show up when the field is larger than 6 T. A distinct peak splitting at the *E*_III_ can be observed with the increase of the magnetic field, and its implication will be discussed later. We use the second derivative of the Landau-level (SDLL) spectra^41^, that is, 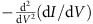, to identify the LL peak positions in further analysis. For instance, the topmost spectrum displays the SDLL spectrum obtained at 11 T.

### Analysis of LLs as a function of magnetic field

**Figure 2:**
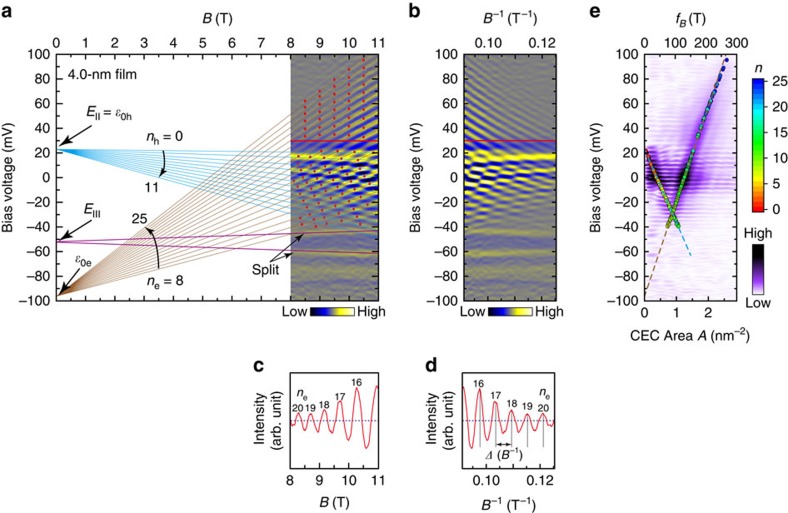
Analysis of the Landau levels. (**a**) The second derivative of the Landau-level (SDLL) pattern of the 4.0-nm-thick film as a function of *B*. Acquisition conditions: −150 mV and 4 nA, modulation of 1.0 mV by root mean square (r.m.s.). The brown and cyan lines are the fan diagrams of the Landau levels (LLs) for the electron-like and hole-like carriers, respectively, extracted from the pattern. The fitted LL indexes (*n*_e_ or *n*_h_), and the extrapolated electron pocket minimum (*ɛ*_0e_) and hole pocket maximum (*ɛ*_0h_) are labelled, where the subscripts ‘e' and ‘h' refer to the electron-like and the hole-like carriers, respectively. The peak splitting at the *E*_III_ is also indicated by the pink lines. (**b**) Replotted SDLL pattern as a function of 1/*B*. (**c**) Profile of the cut-line at 30 mV in **a**. (**d**) Profile of the cut-line at 30 mV in **b**, showing the periodicity of Δ(*B*^−1^). (**e**) Fourier transform pattern of oscillation frequency *f*_*B*_ (top-coordinate) or constant energy cross-sectional (CEC) area *A* (bottom-coordinate), superposed with colour circles that are obtained from the selected points (red dots in **a**) of LL peaks using the Onsager relation. The dashed lines (brown and cyan) in **e** are linear fits of the data (colour circles) for the electron pocket and the hole pocket, respectively. The colour bars in **e** indicate the LL index of the calculated points using the selected points in **a** and the relative intensity of the Fourier transform pattern, respectively.

The SDLL pattern for the 4.0-nm-thick film is shown in Fig. 2a, where the field is swept from 8.0 to 11.0 T with the step of 0.02 T. We can extract two sets of fan diagrams from the overlapped SDLL pattern. According to their positive/negative slopes (brown and cyan lines in Fig. 2a), the fan diagrams are associated with the electron-like or hole-like carriers, respectively. The linearly field-dependent LL peaks in each set are almost equally spaced, suggesting parabolic-like dispersions of the carriers. Therefore, the LLs can be approximately described by *E*_*n*_=*ɛ*_0_±(*n*+*γ*)*ℏω*_c_, where *E*_*n*_ is the energy position of the *n*th LLs, *n* the LL index, *ɛ*_0_ the energy minimum of EP or maximum of HP, *γ* the Onsager phase, *ℏ* the reduced Plank constant, *ω*_c_ the cyclotron frequency, that is, *ω*_c_=*eB*/m*, *e* the electron charge, *B* the magnetic field strength, and m* the cyclotron effective mass of carriers. Signs ‘+' and ‘−' are applied to the electron-like or the hole-like carriers, respectively.

Analogous to the traditional measurements of Shubnikov-de Haas oscillations, the tunnelling magnetoconductance oscillations^42^ can be obtained at every bias voltage, *V*_b_. Figure 2c gives an example of the aperiodic oscillations in the cut-line at +30 mV, where the peaks correspond to the same constant energy cross-sectional area (CEC area, *A*), following the Onsager relation *A*=(2*πe*/*ℏ*)(*n*+*γ*)*B*. The LL indexes for the HP are easily obtained by adopting the topmost LL as *n*_h_=0. In the determination of the LL indexes for the EP, we use the Onsager relation to fit the LL index *n*_e_. At a given energy, for example, see the cut-line at 30 mV in Fig. 2c, the LL peaks correspond to the same value of *A*. The LL indexes fitted by the least square method are obtained and labelled in Fig. 2a. The fitting value of *γ* is about 1/2 and slightly dependent on energy. Tentatively assuming the parabolic dispersion for the EP and HP, we have *eV*_b_−*ɛ*_0_*=*±*ℏ*^2^*k*^2^/2 m*, where *k* is the wave vector. In the momentum space, *A=πk*^2^. Then, we have *A*=±(2πm*/*ℏ*^2^)(*eV*_b_−*ɛ*_0_), which describes the linear dependence of the CEC area *A* on energy, *eV*_b_.

Similar to the analysis used in Shubnikov-de Haas oscillations, the SDLL pattern is replotted as a function of 1/*B* (Fig. 2b), and the oscillations in the cut-line now give the periodicity of Δ(*B*^−1^) (Fig. 2d). The oscillation frequency, *f*_*B*_=1/Δ(*B*^−1^), is obtained by Fourier transform (FT) of every cut-line, and associated with the CEC area by *A*=(2*πe/ℏ*)*f*_*B*_. The FT pattern of the 1/*B* plot is shown in Fig. 2e, which well reflects the linear dependence between *A* and *V*_b_ both for the LLs of the EP and HP, respectively. Alternatively, using the field strength of the marked points at the LL peaks (red dots Fig. 2a), the CEC areas can be obtained by adopting the LL indexes using the Onsager relation. In the overlapped region of the SDLL pattern we have selected the cross points that belong to the LLs both from the EP and HP. For the LLs above 40 mV (only from the EP), the points at the magnetic field strength of 8.5, 9.0, 9.5, 10.0, and 10.5 T are chosen, respectively. The CEC areas obtained from the individual points are superposed in Fig. 2e, denoted by coloured circles. Obviously, these data from the EP and HP can be well-fitted linearly, respectively, shown by the dashed lines in brown and cyan.

### Comparing DFT calculations with experimental results

**Figure 3:**
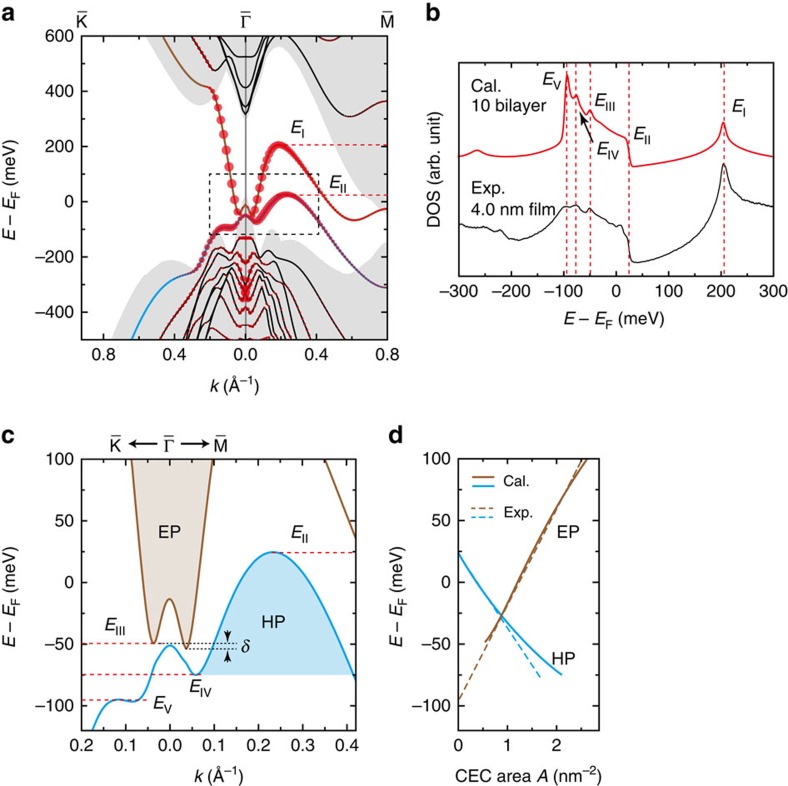
Calculated electronic structure of Bi (111) surface. (**a**) Calculated band structure (solid lines) of Bi (111) surface using a model of 10 Bi bilayers. The surface-state weightings are denoted by the relative size of the red dots. The shadow background is the calculated projection of bulk band structure. The calculated electronic structure is shifted downward by 18 meV for comparing with the experimental results. (**b**) Calculated density of states (DOS, solid red curve) with a Gaussian broadening of 3.0 meV, in comparison with the experimental d*I/*d*V* spectrum (in black) of the 4.0-nm-thick film. (**c**) Magnified band structure around the 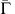 point of the dashed rectangle region in **a**. The dashed red lines correspondingly indicate the energy positions of *E*_*i*_ (*i*=I, II…, V), and the dashed black lines in **c** indicate the depth, *δ*, of the additional tiny pockets with respect to the *E*_III_. (**d**) Calculated constant energy cross-sectional (CEC) areas of the electron pocket (EP) and the hole pocket (HP) against energy, given by the solid lines in brown and cyan, in comparison with the fitted lines from the experimental data of the 4.0-nm-thick film, given by the dashed lines in brown and cyan.

Our experimental observations can be well interpreted by the electronic structures of the Bi (111) surface obtained from the DFT calculations. Figure 3a shows the calculated band structure using a thin-film modelled with 10 Bi bilayers, corresponding to a 4.0-nm-thick film. The two branches in brown and cyan dominantly contribute to the surface states. Figure 3b gives the calculated density of states from these two branches, which well describes the experimentally observed five features in the d*I*/d*V* spectra. For comparison with the experimental results, we set the *E*_F_ to +18.0 meV, that is, the calculated electronic structure is shifted downwards by 18 meV. Except the step-like feature at the *E*_II_ (HP maxima, *ɛ*_0h_), other features appeared as peaks are from the van Hove singularities (VHSs)^43^, that is, the saddle points (*E*_I_, *E*_III_ and *E*_IV_) or the plateaus (*E*_V_) in the band structure, as shown by the magnified surface-state band structure in Fig. 3c. This band structure can well explain the overlap of the two LL sets in our experiment. The EP does not drop to the extrapolated minimum (*ɛ*_0e_), but forms a Mexican-hat-like bottom around −50 meV. Additional tiny pockets, with a depth of *δ*∼4 meV with respect to the *E*_III_, can be seen at the EP bottom in the 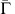
→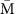 directions. It is noted that the EP and HP are both deviated from the ideal parabolic dispersion, appearing as hexagonal and ellipse-like shapes. In comparison, the calculated Fermi contour is in good accordance with the ARPES results from Bi crystal and Bi films^23^,^24^,^25^,^26^,^27^ (Supplementary Fig. 1, Supplementary Note 1). The CEC areas of the energy-overlapped EP and HP are calculated and shown in Fig. 3d, which are in good agreement with our experimental results.

### Dependence of the surface states on film thickness

**Figure 4:**
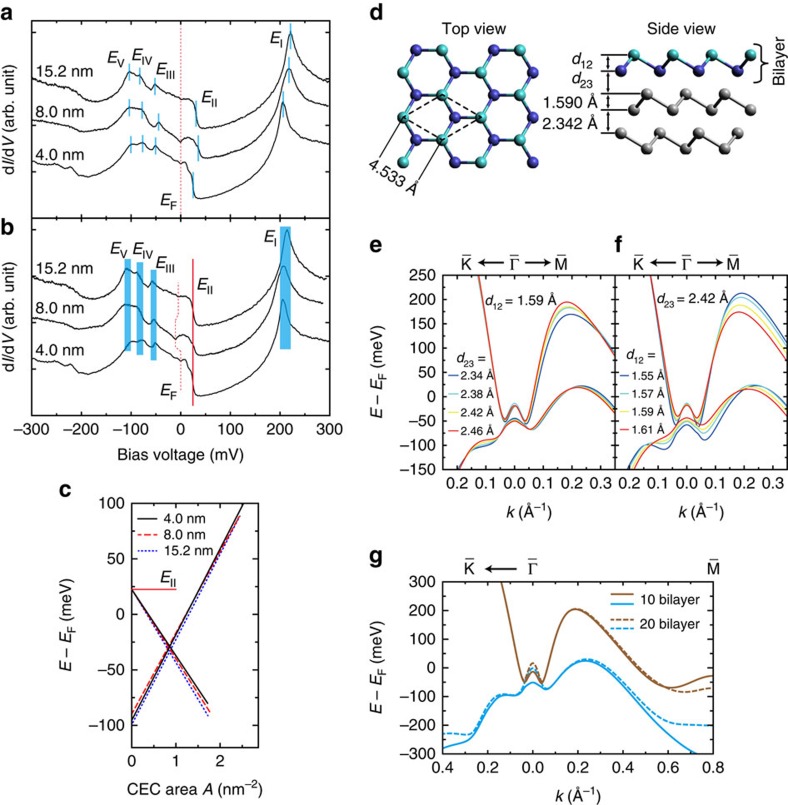
Dependent aspects of the surface states. (**a**,**b**) Original and aligned spectra obtained from films with different thicknesses, showing that the relative intervals of the features are just slightly dependent on the film thickness. The spectra in **b** are aligned according to the *E*_II_ of the 4.0-nm-thick film, as labelled by solid red line. The *E*_F_ is marked by the dashed red lines. The energy positions of other features are marked by solid cyan lines (before alignment in **a**) and cyan bars (after alignment in **b**). The spectra in **a** and **b** are shifted vertically for clarity. (**c**) Constant energy cross-sectional (CEC) areas obtained from fitting the Landau levels of the samples with different thicknesses, respectively, aligned according to the *E*_II_ of the 4.0-nm-thick film. (**d**) Structural model and parameters used in the calculations. (**e**,**f**) Calculated surface-state band branches by fixing *d*_12_ but varying *d*_23_, and by fixing *d*_23_ but varying *d*_12_, respectively, for the terminated Bi bilayer. (**g**) Calculated surface-state band branches of 10 (solid lines) and 20 (dashed lines) Bi bilayers.

It has been theoretically predicted that the surface-state band structure of Bi films has just a slight dependence on the thickness when the films are thicker than 10 bilayers^39^. The typical d*I*/d*V* spectra taken from several samples with different thicknesses at 0 T are shown in Fig. 4a, and replotted in Fig. 4b by aligning them according to the *E*_II_ of the 4.0-nm-thick film. It is observed that the main features in d*I*/d*V* may shift upward or downwards with respect to the *E*_F_ in different samples and even in different sites of the same sample (Supplementary Figs 2–4). Such shifts are attributed to the variations of the Fermi energy in different samples or even local sites affected by the STM tip^44^. Despite the energy shift the intervals between the features just slightly vary, indicating their surface-dominant nature and weak dependence on film thickness. Figure 4c shows the CEC areas obtained from the LL measurements for the films with different thicknesses (Supplementary Fig. 5), which also reflects the almost independent behaviours of the EP and HP on film thickness. Considering the shift of the *E*_F_ in different samples, we plot the fitting lines by aligning them according to the *E*_II_ of the 4.0-nm-thick film. It was recently reported the existence of semimetal-to-semiconductor transition in thicker film of about 180 bilayers^45^, however, it is quite doubted by just measuring the bands at 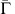 point without having detailed information from the 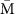 point if the *E*_F_ shift is taken into consideration. Actually, in ref. 45 the changes of the measured CEC areas for the EP and HP in different thicknesses can be well explained by the *E*_F_ shift (Supplementary Table 1).

In our calculations, we used the lattice parameters of bulk crystal with a rhombohedral A7 structure^40^ for the inner Bi bilayers, which gives the distance of 1.590 Å between the atom layers of each bilayer and the distance of 2.342 Å between the neighbour bilayers along the (111) direction, as shown in Fig. 4d. We find that the band structure is much sensitive to the lattice parameters of the terminated Bi bilayer, as shown in Fig. 4e,f. We adopt the parameters of *d*_12_=1.570 Å and *d*_23_=2.420 Å in the terminated Bi bilayer, for these parameters give consistent calculated results with our experimental observations.

We also compared the calculated surface-state bands of 20 bilayers with the ones of 10 bilayers, as shown in Fig. 4g. It is seen that except the states around 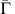, 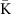 and 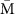 points the surface-state bands of the two films are almost overlapped, which support the observed thickness-independent features. The non-overlapped states around the high symmetric points are ascribed to the hybridization of the quantum well states (QWSs)^26^,^46^.

### Zeeman splitting of spin states at the *E*_III_

**Figure 5:**
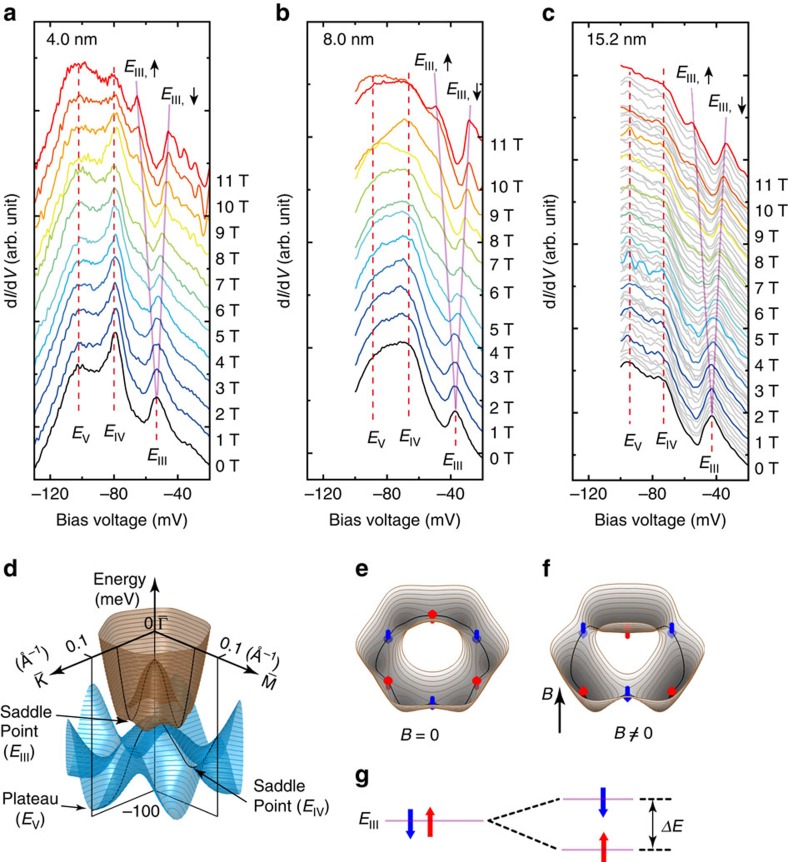
Spin splitting of the surface states at the *E*_III_. (**a**–**c**) d*I*/d*V* spectra of Bi (111) films with thicknesses of 4.0, 8.0, and 15.2 nm, obtained under the labelled magnetic field strengths. Acquisition conditions: −150 mV and 4.0 nA and modulation of 0.5 mV by root mean square (r.m.s.) in **a**, −100 mV and 1.0 nA and modulation of 1.0 mV (r.m.s.) in **b**, and −100 mV and 2.0 nA and modulation of 1.0 mV (rms) in **c**. The spectra in grey are shown with an interval of 0.2 T in **c**. The peak splitting at the *E*_III_ is marked by the pink lines, but the features at the *E*_IV_ and *E*_V_ do not have obvious splitting (dashed red lines). The spectra are shifted vertically for clarity. (**d**) Three-dimensional plot of the band structure around the 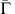 point, with labelled saddle points at the *E*_III_ and *E*_IV_ and the plateau points at the *E*_V_. (**e**,**f**) Schematic drawing of the out-of-plane spin states at the saddle points of *E*_III_ in the momentum space at *B*=0 T, and the spin splitting at *B*≠0 T. (**g**) Energy diagram of the spin degeneracy of the *E*_III_ at *B*=0 T and the spin splitting of Δ*E* at *B*≠0 T. The red and blue arrows denote spin-up and spin-down, respectively.

We observed the peak splitting at the *E*_III_ in all of the three films, as shown in Fig. 5a–c. The peak splitting is attributed to the Zeeman coupling under non-zero magnetic field perpendicular to the surface, which strongly suggests the existence of large out-of-plane spin components at the saddle points of *E*_III_ along the 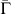
→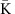 directions (Fig. 5d). Because of the spin texture in the Bi (111) surface, the out-of-plane magnetic moments in two adjacent saddle points should be inversely oriented^21^ (Fig. 5e,f), similar to other related systems^47^,^48^,^49^. At 11 T, the splitting energy, Δ*E*, is about 21 meV in all of the three samples, as illustrated in Fig. 5g. Using Δ*E*=*g*_eff_*μ*_B_*B*, where *μ*_B_=*eℏ*/2*m*_0_ is the Bohr magneton and *m*_0_ is the rest mass of electron, we get an almost thickness-independent effective g-factor, *g*_eff_≈33±1, which is about half of the value for the hole-like carriers in bulk bismuth^50^. This relatively large *g*-factor could be related to the strong SOC^19^ and the *E*_III_ VHS states locating at the turning points from the surface states to the QWSs^26^,^46^. As a result of the Zeeman splitting, spin-polarized valleys may even be produced at alternate *E*_III_ VHS states when the states are deeper than the minima of the tiny pockets at a high enough magnetic field (Fig. 5f). The much large *g*-factor may guarantee the valleys deeper enough over the depth of *δ*. It is noticed that the VHS states at the *E*_III_ have well determined energy and momentum. We did not observe the Zeeman splitting in individual LLs, unlike the observation of the Zeeman splitting of LLs in other 2D system^51^ and in bulk Bi^11^,^50^,^52^. This can be understood by considering that the top and the bottom surfaces of the thin films should have degenerate opposite spin vectors^22^,^53^, while the STM measurements can just detect the top surface of the films.

## Discussion

From the experimentally measured CEC areas (Fig. 4c), we get the estimated electron- and hole-carrier densities to be *D*_e_=3.13 × 10^12^ and *D*_h_=5.82 × 10^12^ cm^−2^ at the *E*_F_ from the 4.0-nm film. In the estimation, we include the sixfold valley degeneracy for HP, but do not include the spin degeneracy both for the EP and HP after considering the fact that the degenerate spin should be separately at the top and the bottom surface in the thin films^22^,^53^. These estimated carrier densities are in good agreement with the previous experimental values of *D*_e_=2.75 × 10^12^ (ref. 54) and *D*_h_=8 × 10^12^ cm^−2^ (ref. 55) obtained in Bi thin films. Such agreements may allow us to suggest that the bottom surface (interface) may not obviously contribute to the conduction, possibly ascribing to the strong scattering at the interface. It is also noticed that our estimated values are just about half of those estimated from the ARPES results by Ast and Höchst^23^. This should be attributed from the inclusion of the spin degeneracy in their calculations, since our measured CEC areas for the EP and HP compare well with the ARPES result (Supplementary Fig. 1, Supplementary Table 1). This observation can be important and useful for the measurement of the 2D electron transport property solely from the top surface in the thin films supported on substrates, which is a highly concerned issue in the study of Bi films^28^,^30^,^31^,^32^,^33^,^34^,^35^,^36^,^56^. In addition, we also expect that Bi (111) thin films may be suitable for the spin transport measurement by using the energy- and momentum-determined spin-polarized *E*_III_ states (Fig. 5).

Using the fitting slopes of the CEC area against the energy (Fig. 4c), we obtain cyclotron effective masses of the electron- and hole-like carriers, *m*_e_*∼(0.16±0.02)*m*_0_ and *m*_h_*∼(0.19±0.03)*m*_0_. It is noticed that in the single crystal Bi (111) surface the ARPES results gave the effective masses of *m*_e_*=(0.22±0.04)*m*_0_ for the electron-like carrier along the 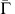
→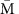 direction^23^, and *m*_hx_*=1.2*m*_0_ along the 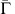
→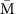 direction and *m*_hy_*=0.032*m*_0_ along the 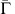
→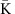 direction for the hole-like carrier^57^. The masses obtained from the LL measurement should reflect the average values over the masses at different *k* points due to the dispersion of the EP and HP deviated from the ideal parabolic dispersion. The effective mass of *m*_e_*∼0.16*m*_0_ in our experiment is still consistent with the value of the single crystal surface, as the EP just slightly deviates from the parabolic dispersion. Due to the ellipse-like cross-sectional shape for HP in momentum space, we get approximately an average value of ∼0.20*m*_0_ by (*m*_hx_*m_hy_*)^1/2^. Obviously, the measured cyclotron effective masses from the LLs in our experiment are consistent with the results from the ARPES measurements.

These relatively large values of the effective masses can be used to explain why we can only observe the LLs under relatively high magnetic fields. The full-width at half maximum (FWHM) of the LL peaks is about 4.5 meV. Since the STM instrumental broadening is smaller than 0.15 meV (Supplementary Fig. 6), the finite-temperature broadening of 3.5*k*_B_*T*∼1 meV, and the used modulation of 0.5 mV (root mean square (r.m.s.)), we can conclude that the observed line width is dominantly contributed by the intrinsic width of LLs. The observed FWHM thus leads to an estimated lifetime of the carriers, *τ*∼0.2 ps. With the criteria *ω*_c_*τ*>1, a lower limit field of about 6 T is obtained. Only above this field can the LL peaks be seen, in agreement with our observations (Fig. 1d, and Supplementary Fig. 5) and the theoretical suggestion^58^. Moreover, the quality of the films is also important to observe the LLs, since the LL peaks may be suppressed when the range of disorder is comparable to the magnetic length *l*_*B*_∼(*ℏ*/*eB*)^1/2^ (ref. 44). Actually, the LLs can be observed at relatively large terraces, but become less pronounced at or near step edges in our experiment (Supplementary Fig. 4). We can also get an estimated carrier mobility of about 1.7 × 10^3^ cm^2^ V^−1^ s^−1^ and a 2D conductivity of 2.4 × 10^−3^ Ω^−1^□^−1^ contributed by the EP and HPs from the Bi (111) top surface. The conductivity is much comparable to the almost temperature-independent conductivity of ∼1.5 × 10^−3^ Ω^−1^□^−1^ obtained in the Bi film of six bilayers^29^,^56^. The slight deviation may be due to the existed scattering by the steps in the films used in the transport measurements^29^,^56^, while our estimation is obtained in a more ideal terrace from the STM measurements.

From our experimental and theoretical results, the EP and HP are quite robust against the change of film thickness from 4.0 to 15.2 nm, even though a certain degree of thickness dependence near the high symmetric points due to the hybridization with QWSs is observed. Considering the fact that the metallic surface states dominantly contribute to the transport properties for films thinner than 20 bilayers^29^, and probably just from the top surface, one may make use of the ultrathin Bi films as a playground to investigate the transport property of multivalley surface states, which possesses huge anisotropic HPs around the *E*_F_. Moreover, the existence of strong out-of-plane spin components at the saddle points of *E*_III_ along the 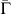
→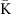 direction can be a novel property to produce spin-polarized valley states. We may thus expect that because of the tunable spin-polarized valleys by magnetic field in addition to the unique surface states, the ultrathin Bi (111) films can indeed find their applications in spintronics and valleytronics^1^,^2^,^3^,^4^,^5^,^6^,^7^.

## Methods

### Sample preparation and characterization

Our STM experiments were conducted using an ultrahigh vacuum (UHV) low temperature scanning tunnelling microscope (UNISOKU) operated under a magnetic field up to 11 T perpendicular to the sample surface, and equipped with a sample preparation chamber for film growth. The base pressure of the system was better than 5 × 10^−11^ torr. The Bi (111) thin films were prepared by depositing Bi (99.997%, MaTeck) with nominal thicknesses of 4.0 and 8.0 nm at a precalibrated rate of 0.12 nm min^−1^ on Si(111)-7 × 7 substrates using a Knudsen cell. During the evaporation, the substrates were kept at room temperature. An As-doped *n*-type Si(111) wafer with resistivity of about 0.001 to 0.005 Ω cm was used. The miscut angle of the Si(111) wafer was about 0.1°, resulting in a Si(111) terrace width of about 200∼400 nm. After the evaporation, the samples were annealed at 380 K for about 24 h. Such treatment helped to produce relatively large terraces in the Bi (111) films. To examine the thicknesses and the corresponding properties, another film with a nominal thickness of about 8.0-nm film was prepared, but followed by annealing at a higher temperature of 420 K for 24 h. This treatment produced islands due to the dewetting of the Bi(111) film^59^. In the islands, the electronic properties of the Bi (111) surface could be well correlated to the determined thicknesses by measuring at the island edges (Supplementary Figs 2 and 3). In this way, the uncertainty of the thicknesses of islands should be within 1 or 2 bilayers. The presented results from the film of 15.2 nm were obtained from a Bi (111) island of about 5 μm. All of the STM measurements were performed at 4.3 K. The d*I*/d*V* spectra of the Bi (111) surface were measured using a lock-in preamplifier, with a sinusoidal modulation of 0.5∼2.0 mV (r.m.s.) at 791 Hz, under various magnetic fields. An electrochemically etched and well-cleaned tungsten tip was used. The sample bias with respect to the tip was used.

The microscope had a thermal drift smaller than 0.5 nm h^−1^ at zero magnetic field at 4.3 K. There was a systematic drift between the tip and the sample about 20 nm with the increase of the magnetic field from 7 to 11 T in our microscope. In the measurements of individual spectra, we always waited for the stabilization of the microscope and repositioned the tip sites when the field was changed. While, in the field-sweeping measurements, we compensated the drift and reexamined the tip sites by every interval of 0.5 T, which could keep the spectra acquired almost at the same sites within an accuracy of about 0.5 nm. For the sake of simplicity, we just focus our discussion of the LL spectra obtained from the centre region of terraces larger than approximately 200 × 200 nm^2^. Each set of the spectra with varied magnetic field were measured almost at the same site by tracing the tip-sample drift, to avoid site-dependent shift of the spectra.

### Theoretical calculations

The calculations of the electronic structure were performed using the all-electron full potential linearized augmented plane-wave method implemented in WIEN2k (ref. 60). The generalized gradient approximation in PBE form^61^ was used to describe the electronic exchange–correlation energy functional. The irreducible surface Brillouin zone (SBZ) was sampled via 12 × 12 × 1 with 144 k points. The muffin tin radius (*R*_mt_) of 2.5 a.u. was used for Bi atom. The plane-wave cutoff parameter *R*_mt_*K*_max_ was chosen to be 9 (*K*_max_ is the magnitude of the largest **K** vector). Inside the muffin tins wave functions were expanded in spherical harmonics up to *l*_max_=12. Spin-orbit coupling (SOC) was included. The Bi (111) surface was modelled by a slab with a (1 × 1) unit cell containing 10 bilayers. A vacuum gap of 15 Å was used between the slabs. The lattice constants of bulk crystal with a rhombohedral A7 structure, that is, *a*=4.533 Å, and *c*=11.797 Å at 4.2 K (ref. 40), were used for the inner Bi bilayers, but the parameters for the outermost bilayer (both at the top and the bottom sides) were adopted by comparing the calculated results with the experimental results.

## Additional information

**How to cite this article:** Du, H. *et al*. Surface Landau levels and spin states in bismuth (111) ultrathin films. *Nat. Commun.* 7:10814 doi: 10.1038/ncomms10814 (2016).

## Supplementary Material

Supplementary Information Supplementary Figures 1-6, Supplementary Table 1, Supplementary Note 1 and Supplementary References
