# Supplementary material for: Surface Landau levels and spin states in bismuth (111) ultrathin films
Source: Nat Commun. 2016 Mar 11;7:10814. doi: 10.1038/ncomms10814 (PMC4792961; doi:10.1038/ncomms10814)
Supplement: Supplementary Information — Supplementary Figures 1-6, Supplementary Table 1, Supplementary Note 1 and Supplementary References [file ncomms10814-s1.pdf]

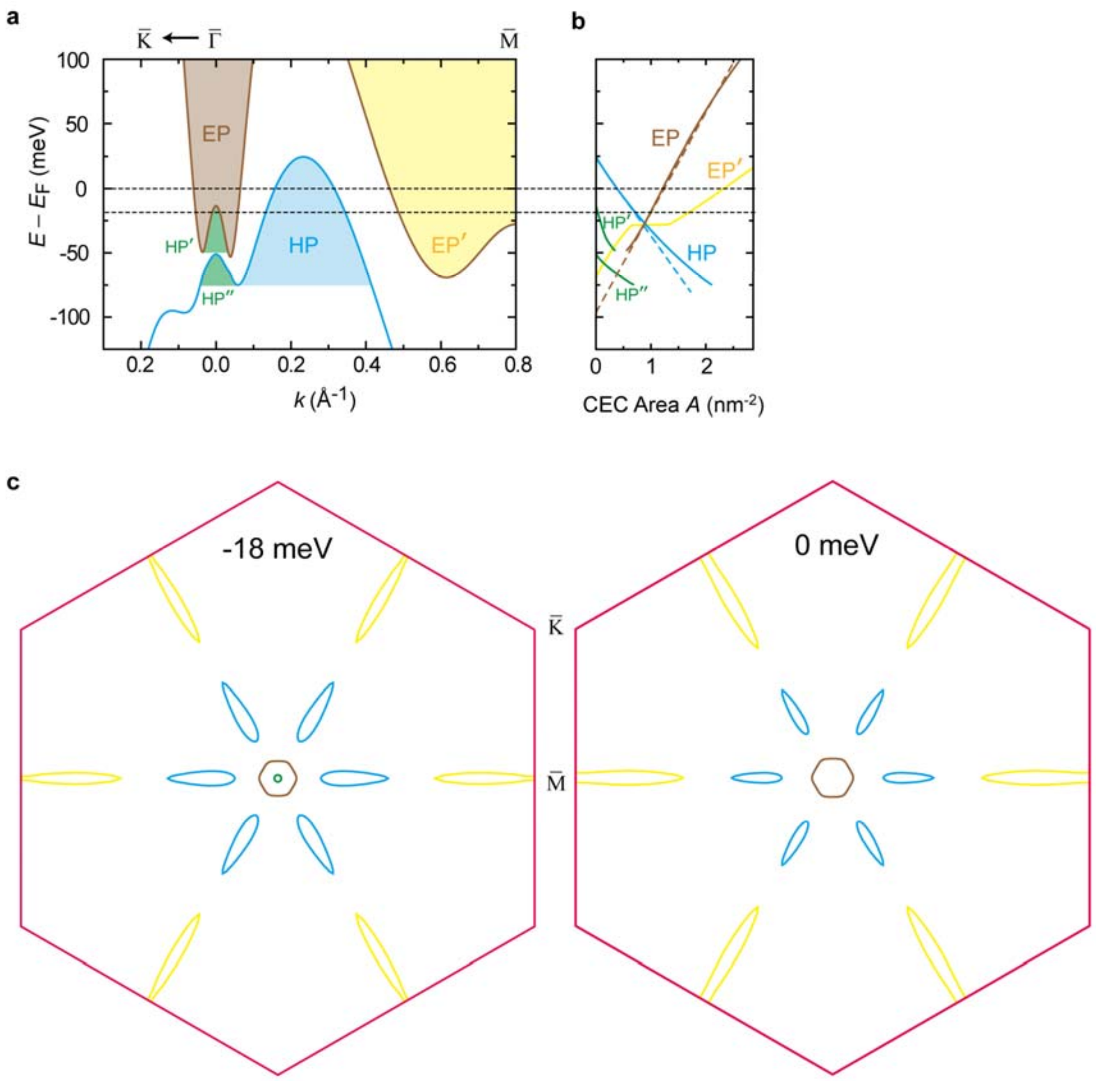

Supplementary Figure 1 | Comparison of calculated Fermi contour with ARPES result. (**a**) Indication of additional electron pockets (EP') around $\overline{\mathrm{M}}$ point, and hole pockets (HP' and HP'') around $\overline{\Gamma}$ point, besides the electron pocket (EP) and the hole pocket (HP). (**b**) Calculated constant energy cross-sectional (CEC) areas in addition to the characterized EP and HP. (**c**) Calculated contour at –18 meV and at the Fermi energy (E_F), where the contour at –18 meV is quite comparable with contour from the angle-resolved photoemission spectroscopy (Supplementary Ref. 1).

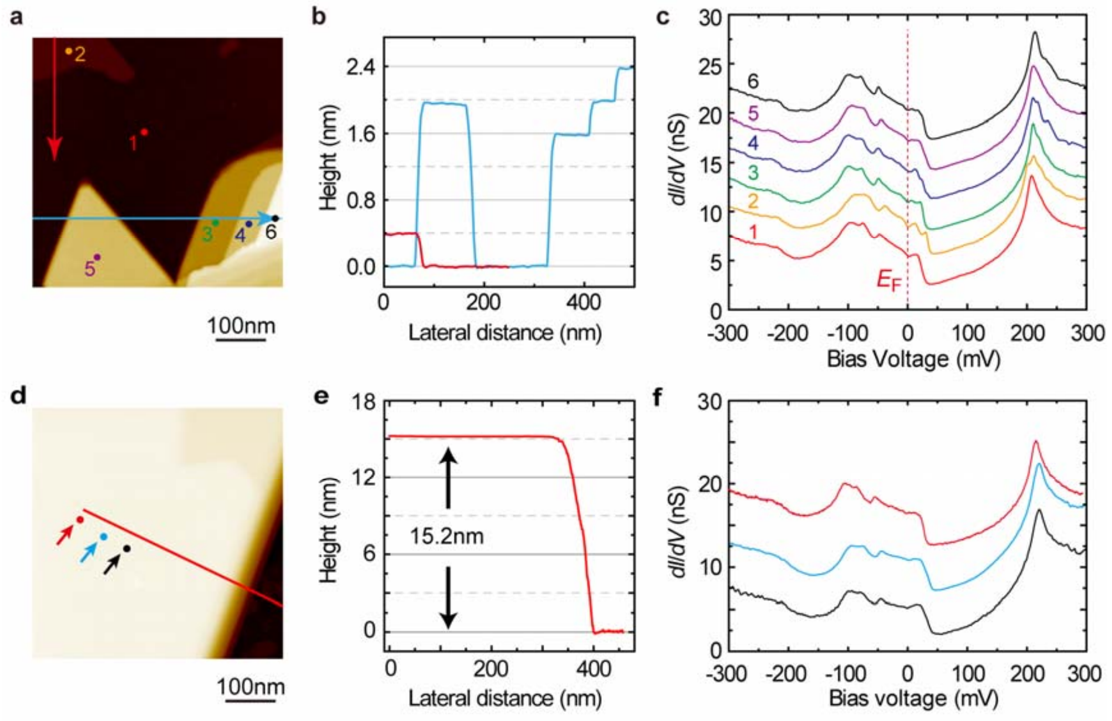

Supplementary Figure 2 | Thickness dependent STS spectra. (**a**-**c**) Measurements of *dI*/*dV* spectra from terraces with different heights in the same sample from an island with a base height of about 4 nm. The spectra in **c** were recorded at the marked sites in **a,** corresponding to different thicknesses of 10~16 bilayers. (**d**-**f**) Measurements of *dI*/*dV* spectra from an island with thickness of 15.2 nm, where the spectra in **f** were recorded at the marked sites in **d**. The images were recorded at –2.0 V and 10 pA. The set points of the spectra are –300 mV and 2 nA with modulation of 1 mV by root mean square (rms). The spectra in **c** and **f** are shifted vertically for clarity.

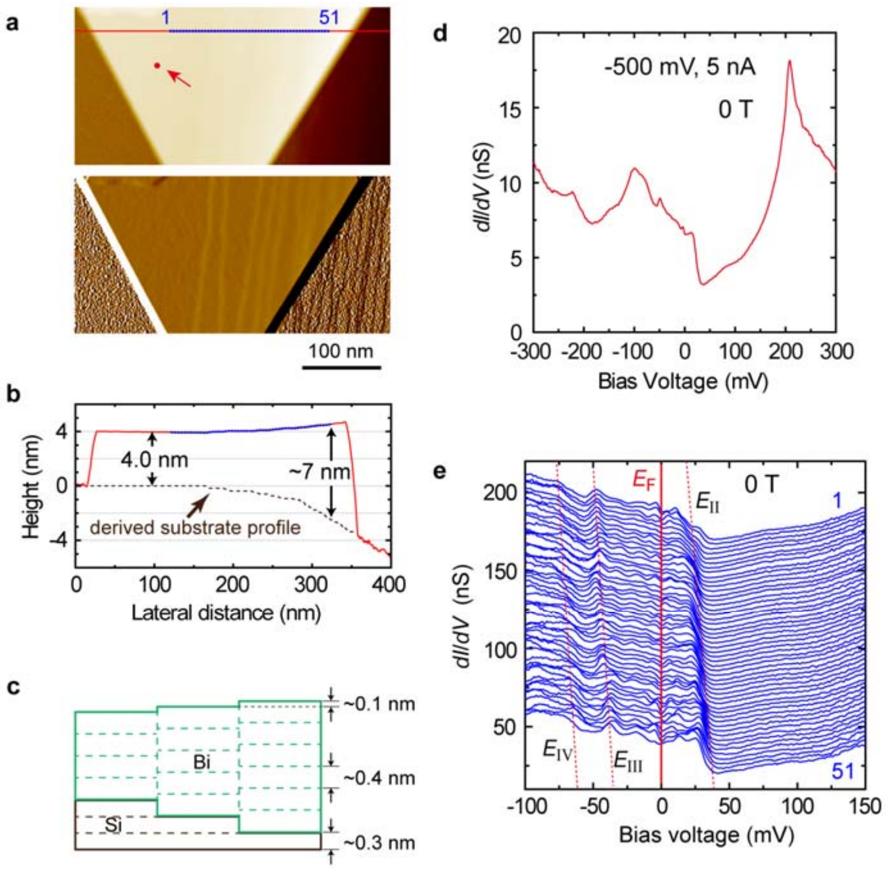

Supplementary Figure 3 | Measurements of the STS spectra in an island. (**a**) STM topographic image (upper) and the corresponding current image (lower) of a Bi (111) island (recorded at –2.0 V and 2 pA). (**b**) Line-profile along the red line in **a**, with a derived substrate profile using the model in **c**. (**c**) Schematic drawing of a Bi (111) film grown a Si (111) substrate with step bunches. (**d**,**e**) *dI*/*dV* spectra acquired at the marked point and along the line shown in **a,** acquired at –100 mV and 5 nA with modulation of 0.5 mV by root mean square (rms). Each spectrum was averaged over 10 repeated measurements. The spectra in **e** are shifted vertically for clarity. The dashed lines are for eyes guide only, showing the nearly unchanged intervals between the features (see, E_{II}, E_{III} and E_{IV}). Measured at zero field.

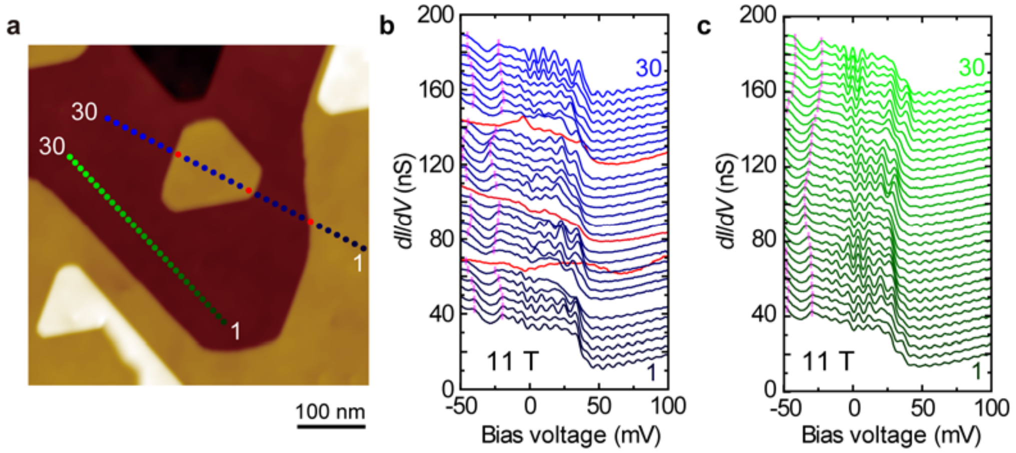

Supplementary Figure 4 | Site-dependent LLs of a 4.0-nm-thick film at 11 T. (**a**) STM image (–1.0 V and 10 pA) showing the acquisition sites of LL spectra. (**b**) Spectra acquired across step edges, and (**c**) spectra acquired within a terrace but with an island nearby. The spectra were acquired at –50 mV and 2 nA with modulation of 1 mV by root mean square (rms) at 11 T field. The dashed pink lines mark the splitting peaks of the E_{III}. The spectra are shifted vertically for clarity.

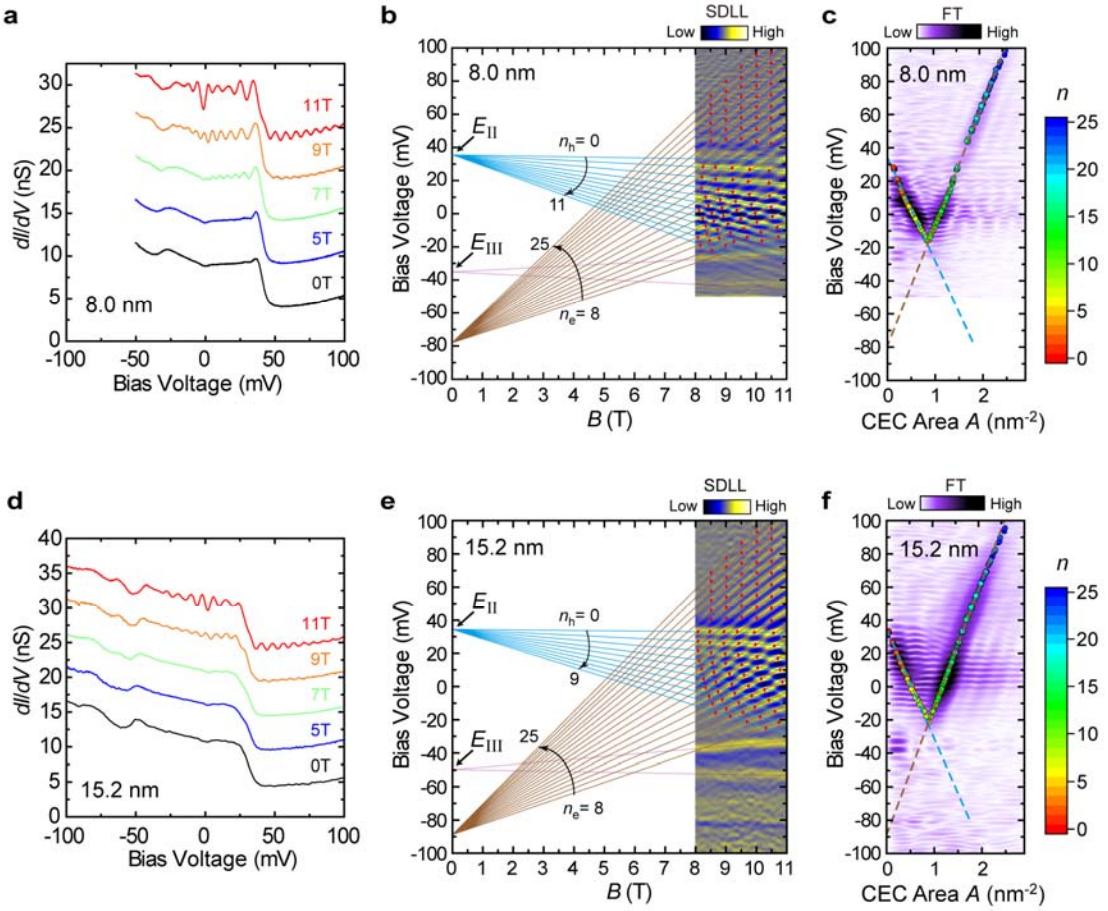

Supplementary Figure 5 | Analysis of the CEC areas for films of 8.0 and 15.2 nm. (**a**-**c**) Measuring the constant energy cross-sectional (CEC) areas for film of 8.0 nm: *dI*/*dV* spectra in **a**, second derivative of the Landau-level (SDLL) pattern in **b**, and CEC areas in **c**, acquired at –50 mV and 0.5 nA with modulation of 0.5 mV (rms). (**d**-**f**) Measuring the CEC areas for film of 15.2 nm: *dI*/*dV* spectra in **d**, SDLL pattern in **e**, and CEC areas in **f**, acquired at –150 mV and 2 nA with modulation of 1.0 mV (rms). The spectra are shifted vertically for clarity. The dashed lines are obtained from linearly fitting to the experimental data in **c** and **f**.

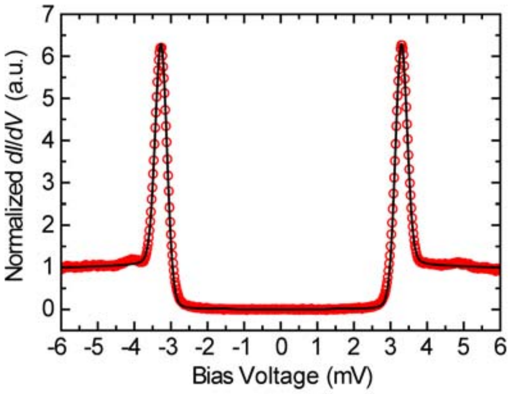

Supplementary Figure 6 | Measurement of the STM energy resolution. *dI*/*dV* spectrum measured on Pb superconductor surface using a Nb tip at 0.43K. The differential conductance at –6 meV was normalized to 1. It gives an instrumental broadening of 0.15 meV by fitting experimental data using a superconductor-insulator-superconductor model, red circles: experimental data, black curve: fitting curve.

Pockets in different samples		0 meV		−18 meV	
		CEC Area (nm^{-2})	Carrier Density $\times 10^{12}$ (cm^{-2})	CEC Area (nm^{-2})	Carrier Density $\times 10^{12}$ (cm^{-2})
4.0 nm	EP	1.24±0.02	3.13±0.05	1.01±0.01	2.55±0.03
	HP	0.38±0.01	5.82±0.15	0.68±0.01	10.38±0.15
8.0 nm	EP	1.23±0.04	3.10±0.10	0.98±0.04	2.48±0.10
	HP	0.37±0.02	5.59±0.30	0.66±0.03	9.97±0.45
15.2 nm	EP	1.30±0.02	3.29±0.05	1.06±0.01	2.70±0.03
	HP	0.35±0.02	5.27±0.30	0.62±0.02	9.40±0.30

Supplementary Table 1 | Estimated electron- and hole-carrier densities.

Supplementary Note 1 | Comparison of calculated surface states with previous ARPES results.

From the calculated band structure, besides the hexagonal EP around the $\overline{\Gamma}$ point and six HPs along the $\overline{\Gamma} \rightarrow \overline{M}$ directions, there are six electron pockets around $\overline{M}$ points (denoted by EP′) and hole pockets around the $\overline{\Gamma}$ point (denoted by HP′ and HP′′), as shown in Supplementary Fig. 1a. The calculated constant energy cross-sectional (CEC) areas of these pockets are shown in Supplementary Fig. 1b, where the jump in CEC area of EP' corresponds to the Lifshitz transition. We do not observe obvious LL signal from the EP′, HP′ and HP′′, which could be due to their relatively small surface-state weightings (Fig. 3a in the main text). We therefore do not include their contributions to the carrier density and conductivity of the surface in our discussion.

We have compared the calculated Fermi contour with those obtained from the Bi (111) surface of single crystal[1-3] and films[4,5] and in previous angle-resolved photoemission spectroscopy (ARPES) measurements. As shown in Supplementary Fig. 1c, the shapes and areas of the EP and HP are quite comparable with the ARPES results even for the single crystal surface[1]. It is noted that the contour is cut at −18 meV along the dashed line in Supplementary Fig. 1a, the Fermi level of the calculated band structure before it is shifted. The shift of the Fermi level is observed to be sample-dependent and even site-dependent in our experiment (Supplementary Figs. 2-4). We have carefully considered this situation and compared the results in different

samples (Supplementary Fig. 5). From our analyses, we believe that such shifts basically do not affect our conclusions. However, the electron- and hole-carrier densities estimated from the CEC areas at –18 meV and at the Fermi energy (E_F) (Fig. 4c in the main text) may vary in a certain range, as given in Supplementary Table 1. In the estimation, the 6-fold degeneracy of HP is considered, but the spin degeneracy is not included for the reason of better comparison of the carrier densities with those previously obtained from transport measurements in Bi films[6,7].

Supplementary References

1. Ast, C. R. & Höchst, H. Fermi surface of Bi(111) measured by photoemission spectroscopy. *Phys. Rev. Lett.* **87,** 177602 (2001).
2. Ast, C. R., & Höchst, H. Indication of charge-density-wave formation in Bi (111). *Phys. Rev. Lett.* **90,** 016403 (2003).
3. Ohtsubo, Y., Mauchain, J., Faure, J., Papalazarou, E., Marsi, M., Le Fèvre, P., Bertran, F., Taleb-Ibrahimi, A. & Perfetti, L. Giant anisotropy of spin-orbit splitting at the bismuth surface. *Phys. Rev. Lett.* **109,** 226404 (2012).
4. Hirahara, T., Nagao, T., Matsuda, I., Bihlmayer, G., Chulkov, E. V., Koroteev, Y. M., Echenique, P. M., Satio, M. & Hasegawa, S. Role of Spin-Orbit Coupling and Hybridization Effects in the Electronic Structure of Ultrathin Bi Films. *Phys. Rev. Lett.* **97,** 146803 (2006).
5. Hirahara, T., Nagao, T., Matsuda, I., Bihlmayer, G., Chulkov, E., Koroteev, Y. M. & Hasegawa, S. Quantum well states in ultrathin Bi films: Angle-resolved

photoemission spectroscopy and first-principles calculations study. *Phys. Rev. B* **75,** 035422 (2007).

6. Komnik , Y. F. & Andrievskii, V. V. Kinetic properties of bismuth thin films. *Sov. J. Low Temp. Phys.* **1,** 51-58 (1975).

7. Hoffman, C. A., Meyer, J. R., Bartoli, F. J., Di Venere, A., Yi, X. J., Hou, C. L., Wang, H. C., Ketterson, J. B. & Wong, G. K. Semimetal-to-semiconductor transition in bismuth thin films. *Phys. Rev. B* **48,** 11431-11434 (1993).
